# Unique compound with anti-allergic action: inhibition of Lyn kinase activity by KIRA6

**DOI:** 10.3389/fphar.2025.1625798

**Published:** 2025-07-25

**Authors:** Goshi Matsushima, Yuri Matsui, Hanano Okamoto, Nagisa Umeda, Mai Kakimoto, Megumi Mino, Tadashi Nakagawa, Kaori Ishii, Yoshimi Matsuo, Daiki Matsubara, Akio Tanaka, Koji Ogata, Michiko Yoshii, Mitsuhiro Goda, Yuhki Yanase, Toru Hosoi, Koichiro Ozawa

**Affiliations:** ^1^Department of Pharmacotherapy, Graduate School of Biomedical and Health Sciences, Hiroshima University, Hiroshima, Japan; ^2^Department of Clinical Pharmacology, Faculty of Pharmaceutical Sciences, Sanyo-Onoda City University, Yamaguchi, Japan; ^3^Department of Dermatology, Graduate School of Biomedical and Health Sciences, Hiroshima University, Hiroshima, Japan; ^4^Faculty of Pharmaceutical Sciences, Sanyo-Onoda City University, Yamaguchi, Japan; ^5^Faculty of Pharmacy, Yasuda Women’s University, Hiroshima, Japan

**Keywords:** allergic disorder, endoplasmic reticulum stress, Lyn, degranulation, mast cell, basophil

## Abstract

Mast cells and basophils play important roles in allergic disorders associated with specific antigens and IgE. Crosslinking of the high-affinity IgE receptor (FcεRI) by specific antigens activates several tyrosine kinases, such as Lyn and spleen-associated tyrosine kinase (Syk), resulting in the release of calcium ions (Ca^2+^) from the endoplasmic reticulum (ER) into the cytoplasm. As Ca^2+^ release from the ER is essential for the release of pro-inflammatory mediators, ER stress-related molecules, such as inositol-requiring enzyme 1α (IRE1α), may play roles in mast cell and basophil activation. However, the associations between ER stress-related molecules and mast cell and basophil activation remain unclear. In this study, we aimed to investigate the roles of ER stress-related molecules in mast cell and basophil activation. Activation of the IRE1α-spliced form of the X-box binding protein 1 (sXBP1) axis, an ER stress-related pathway, was observed during the antigen-induced activation of mast cells. Moreover, the IRE1α inhibitor, KIRA6, suppressed antigen-induced release of pro-inflammatory mediators from rat basophilic leukemia (RBL)-2H3 cells, bone marrow-derived mast cells (BMMCs), human basophils, and human mast cells at low doses (<1 μM). However, to our surprise, IRE1α knockout did not inhibit antigen-induced release of pro-inflammatory mediators. Instead, KIRA6 blocked the antigen-induced activation of Syk by inhibiting kinase activity of Lyn. Additionally, KIRA6 exerted anti-allergic effects *in vivo*. Overall, our findings suggest that KIRA6 prevents allergic reactions by inhibiting the kinase activity of Lyn via an IRE1α-independent pathway.

## Introduction

Allergic diseases affect a large number of individuals and significantly impair their quality of life. However, the anti-allergic effects of existing drugs are often insufficient. Therefore, effective anti-allergic agents with mechanisms of action different from those of conventional drugs are clinically required. Although endoplasmic reticulum (ER) stress is related to various disorders (Liu et al., 2024), the role of ER stress-related proteins, such as inositol-requiring enzyme 1α (IRE1α), double-strand RNA-dependent protein kinase-like ER kinase (PERK), and activating transcription factor (ATF)-6, for the induction of allergic reactions has not been clarified.

Mast cells resident in tissue and basophils circulating in blood vessels play important roles in antigen (allergen)- and IgE antibody-associated allergic disorders, such as asthma, urticaria, pollen allergy, allergic rhinitis, and food allergy (Siracusa et al., 2013; Amin, 2012). They express the high-affinity IgE receptor (FcεRI) on their surface (Shamji et al., 2021; Galli et al., 2008). Crosslinking of FcεRI on their cell surface by IgE antibodies and specific antigens induces the activation of several tyrosine kinases, such as Lyn and spleen-associated tyrosine kinase (Syk), followed by the activation of phospholipase C (PLC), which produces inositol trisphosphate (IP_3_) from phosphatidylinositol 4,5-bisphosphate (PIP_2_). IP_3_ further induces the release of calcium ions (Ca^2+^) from the ER via the IP_3_-sensitive channel of the ER (Li et al., 2022). Increased intracellular Ca^2+^ levels activate various downstream proteins, leading to the rapid release of stored mediators, such as histamine, from secretory granules (degranulation).

Subsequently, newly synthesized mediators, including arachidonic acid metabolites such as leukotriene C4 (LTC4) and pro-inflammatory cytokines such as interleukin (IL)-6 and tumor necrosis factor (TNF)-α, are also released within a few hours. These molecules activate various targets, such as vascular endothelial cells, smooth muscles, and sensory neurons, resulting in allergic reactions (Galli et al., 2008; Blank et al., 2021; Sarin et al., 2006; Hammad and Lambrecht, 2008). Many studies have investigated the mast cell and basophil activation mechanisms; however, potent compounds suppressing mast cell and basophil functions have not yet been developed.

As Ca^2+^ release from the ER is a critical event for mast cell and basophil activation, ER stress-related molecules possibly regulate mast cell and basophil functions. ER maintains protein homeostasis by regulating protein synthesis, processing, and transport. Accumulation of unfolded proteins in the ER in response to various internal and external stimuli induces ER stress, resulting in apoptosis (Hosoi and Ozawa, 2009; Walter and Ron, 2011; Wang and Kaufman, 2016; Lindholm et al., 2006). The ER has three major sensor proteins for ER stress: IRE1α, PERK, and ATF-6. Upon detecting ER stress, these proteins activate the unfolded protein response (UPR), which involves IRE1α-mediated induction of X-box binding protein 1 (XBP1) mRNA splicing (sXBP1; the activated form of XBP1), CCAAT/enhancer-binding protein homologous protein (CHOP), a pro-apoptotic transcription factor, and glucose-regulated protein 78 (GRP78), a prominent ER-resident molecular chaperone and key regulator of the ER stress response (Walter and Ron, 2011; Wang and Kaufman, 2016; Ron and Walter, 2007; Zhang and Kaufman, 2008). ER stress is also induced by Ca^2+^ depletion in the ER (Sehgal et al., 2017; Pontisso et al., 2024). Therefore, antigen-induced Ca^2+^ mobilization in mast cells and basophils possibly activates the ER stress-related molecules. Sehgal et al. (2017) reported that the inhibition of sarco/ER Ca^2+^–ATPase by thapsigargin (Tg) analogs induces ER Ca^2+^ depletion and UPR. Moreover, Pontisso et al. (2024) reported that the decrease in ER Ca^2+^ levels by the Ca^2+^–ATPase inhibitor, 2,5-di-(tert-butyl)-1,4-benzohydroquinone (tBuBHQ), induces IRE1α activation, suggesting that antigen-induced Ca^2+^ release from the ER in mast cells and basophils activates the IRE1α–sXBP1 axis.

In this study, we investigated the roles of IRE1α and the effects of its inhibitors, such as KIRA6, on the allergic actions of mast cells and basophils.

## Materials and methods

### Reagents

Anti-dinitrophenyl (DNP) IgE antibodies, DNP–bovine serum albumin (BSA), BSA, p-nitrophenyl-N-acetyl-β-glucosamine, Ceapin-A7, and Tg were purchased from Sigma-Aldrich (Tokyo, Japan). GSK2606414 was purchased from LKT Laboratories (St. Paul, MN, United States). APY29 was purchased from AdooQ Bioscience (Irvine, CA, United States). Additionally, 4μ8c was obtained from Merck (Darmstadt, Germany). Anti-IgE antibodies were purchased from Bethyl Laboratories (Montgomery, TX, United States). Recombinant mouse IL-3 and human stem cell factor were purchased from R&D Systems Inc. (Minneapolis, MN, United States). Penicillin/streptomycin and trypsin were purchased from Life Technologies (Carlsbad, CA, United States). Roswell Park Memorial Institute (RPMI)-1640 medium, Hank’s balanced salt solution (HBSS), and 2-mercaptoethanol were obtained from Nacalai Tesque (Kyoto, Japan). KIRA6 was obtained from Selleck Biotech (Kanagawa, Japan). KIRA8 and amlexanox were purchased from MedChemExpress (Monmouth Junction, NJ, United States). Tranilast and epinastine were purchased from Tokyo Chemical Industry (Tokyo, Japan).

### Rat basophilic leukemia-2H3 mast cell culture

Rat basophilic leukemia (RBL)-2H3 cells were cultured in the RPMI-1640 medium supplemented with 10% fetal bovine serum (FBS) (Nichirei, Tokyo, Japan), 100 IU/mL penicillin G, and 100 μg/mL streptomycin.

### Isolation of bone marrow-derived mast cells

Bone marrow-derived mast cells (BMMCs) were obtained from 8–10-week-old BALB/c female mice (Charles River Laboratories, Yokohama, Japan), as previously described (Okabe et al., 2006). In brief, bone marrow cells were suspended at a density of 1 × 10^6^ cells/mL in the RPMI-1640 medium supplemented with 10% FBS, 50 μM 2-mercaptoethanol, 2 mM glutamine, 100 IU/mL penicillin G, 100 μg/mL streptomycin, and 5 ng/mL IL-3 for 4 weeks.

### Detection of sXBP1

Total RNA was extracted using Sepasol-RNA I Super G (Nacalai Tesque, Kyoto, Japan). Then, cDNA was synthesized from 0.2 μg (BMMCs) or 2 μg (RBL-2H3 mast cells) of total RNA via reverse transcription using ReverTra Ace (Toyobo, Osaka, Japan) and Oligo (dt)_16_ primer (Eurofins Genomics, Tokyo, Japan) in a 20-μL reaction mixture containing RT buffer (Toyobo), 1 mM dNTP mix (Toyobo), and 20 U of the RNase inhibitor (Enzymatics, Beverly, MA, United States). Total RNA and Oligo (dt)_16_ primer were pre-incubated at 70 °C for 10 min prior to reverse transcription. After incubation for 1.5 h at 46 °C, the reaction was terminated by incubating the samples at 100 °C for 5 min. For polymerase chain reaction (PCR) amplification, 1.2 μL of cDNA was added to 10.8 μL of reaction mix containing 1 μM of each primer, KAPA Taq EXtra Buffer, 0.3 mM of KAPA dNTP mix, 1.75 mM of MgCl_2_, and 0.3 U of KAPA Taq EXtra DNA polymerase (Roche, Basel, Switzerland). The following primer sequences were used: m(r)glyceraldehyde 3-phosphate dehydrogenase (GAPDH) upstream 5′-AAA​CCC​ATC​ACC​ATC​TTC​CAG-3′, m(r)GAPDH downstream 5′-AGG​GGC​CAT​CCA​CAG​TCT​TCT-3′, rXBP1 upstream 5′-CTT​GTG​ATT​GAG​AAC​CAG​GAG-3′, rXBP1 downstream 5′-AAG​AGG​CAA​CAG​CGT​CAG-3′, mXBP1 upstream 5′-CCT​TGT​GGT​TGA​GAA​CCA​GG-3′, and mXBP1 downstream 5′-CTA​GAG​GCT​TGG​TGT​ATA​C-3′. PCR products (10 μL) were resolved via electrophoresis on an 8% polyacrylamide gel. The gels were stained with ethidium bromide and photographed under ultraviolet light. The density of each band was measured using ImageJ 1.37v software (National Institutes of Health, MD, United States).

### Degranulation assay

Degranulation was assessed by measuring the release of β-hexosaminidase, a granule marker that hydrolyzes *p*-nitrophenyl-*N*-acetyl-β-glucosamine to the chromophore, *p*-nitrophenol, as previously described (Hide et al., 1997). In brief, RBL-2H3 mast cells, BMMCs, or human skin mast cells (hsMCs) sensitized with anti-DNP IgE antibodies overnight were resuspended in piperazine-*N*- *N*′-bis (2-ethanesulfonic acid) (PIPES) buffer containing 119 mM NaCl, 5 mM KCl, 1.0 mM CaCl_2_, 0.4 mM MgCl_2_, 5.6 mM glucose, 25 mM PIPES, and 1 mg/mL BSA (pH 7.2). After incubation with or without inhibitors for 30 min at 37 °C, the cells were stimulated with 100 or 50 ng/mL DNP-BSA antigens or 670 ng/mL anti-IgE antibodies at 37 °C for 15 min.

### Culture of human skin mast cells

hsMCs were isolated from human skin, as previously described (Yanase et al., 2021). In brief, the skin was cut into fragments and incubated in HBSS containing type 2 collagenase (1.5 mg/mL), hyaluronidase (0.7 mg/mL), type I DNase (0.3 mg/mL), 1% FCS, and 1 mM CaCl_2_ at 37 °C for 2 h. The dispersed cells were suspended in the X-VIVO-15 medium (Lonza, Walkersville, MD, United States). Isolated mast cells were cultured in the X-VIVO-15 medium containing 100 ng/mL recombinant human stem cell factor for 1–2 months.

Informed consent was obtained from four independent donors according to the ethical standard of Hiroshima University (approval number: E2014-1115).

### Isolation of peripheral blood mononuclear cells from human peripheral blood

Human peripheral blood mononuclear cells (PBMCs) were isolated from the fresh heparinized blood of drug-free healthy donors via Ficoll-Paque Plus density gradient separation.

Informed consent was obtained from four independent donors according to the ethical standards of Hiroshima University (approval number: E2019-1716).

### Histamine release test

Histamine release tests using human peripheral blood basophils were performed with goat anti-IgE antibodies as a positive control, as previously described (Matsuo et al., 2018). Histamine was extracted and measured using reverse-phase high-performance liquid chromatography (HPLC).

### Quantitative PCR analysis

Two-step quantitative PCR (qPCR) was performed using the Brilliant III Ultra-Fast SYBR Green qPCR Master Mix (Agilent Technologies, Santa Clara, CA, United States) and PikoReal 96 (Thermo Fisher Scientific, Waltham, MA, United States), according to the manufacturers’ protocols. The cycling protocol was as follows: DNA polymerase activation at 95 °C for 5 min, followed by denaturation at 95 °C for 5 s and annealing/extension at 60 °C for 20 s for 40 cycles. The relative level in each sample was normalized to the GAPDH mRNA level in the same sample and calculated using the ΔΔCT analysis. qPCR was performed using the following primers: mIL-6 upstream 5′-GTT​CTC​TGG​GAA​ATC​GTG​GA-3′, mIL-6 downstream 5′-TGT​ACT​CCA​GGT​AGC​TAT​GG-3′, mTNF-α upstream 5′-CAC​GTC​GTA​GCA​AAC​CAC​CAA-3′, mTNF-α downstream 5′-CCC​ATT​CCC​TTC​ACA​GAG​CAA-3′, mGAPDH upstream 5′-TGC​ACC​ACC​AAC​TGC​TTA​GC-3′, mGAPDH downstream 5′-GGC​ATG​GAC​TGT​GGT​CAT​GAG-3′, rat TNF-alpha qPCR primer pair (Sino Biological Inc., Beijing, China), rat IL-6 qPCR primer pair (Sino Biological Inc.), rGAPDH upstream 5′-AAA​CCC​ATC​ACC​ATC​TTC​CAG-3′, and rGAPDH downstream 5′-AGG​GGC​CAT​CCA​CAG​TCT​TCT-3′.

### Enzyme-linked immunosorbent assay

Enzyme-linked immunosorbent assay (ELISA) was performed using Mouse TNF-α DuoSet ELISA (R&D Systems), the LEGEND MAX Mouse IL-6 ELISA Kit (BioLegend, San Diego, CA, United States), and the Leukotriene C4 ELISA Kit (Cayman, Ann Arbor, MI, United States), according to the manufacturers’ protocols.

### Generation of IRE1-knocked-out RBL-2H3 mast cells

The CRISPRdirect design tool (Naito et al., 2015) was used to identify the optimal target sequences of SpCas9 for the rat *Ern1* gene, which encodes IRE1α. Spacer-coding DNAs was inserted into lentiCRISPRv2 (#52961; Addgene, Watertown, MA, United States) using the BsmBI cloning site to generate lentiCRISPRv2-rEnr1-1 or lentiCRISPRv2-rEnr1-2 (Sanjana et al., 2014). Protospacer sequences were 5′-ATG​CAA​ACT​TCC​GTC​CAG​GG-3′ (KO1) and 5′-AGA​GGA​CAG​GCT​CCA​TCA​AG-3′ (KO2). Lentivirus was produced by co-transfecting 2 × 10^6^ HEK-293T cells in a 10-cm dish with 2 μg of lentiCRISPRv2-rEnr1-1 or lentiCRISPRv2-rEnr1-2, 2 μg of psPAX2 (#12260; Addgene), and 2 μg of pMD2.G (#12259; Addgene) using 25 μg of PEI MAX (Polysciences, Warrington, PA, United States). Twenty-four hours post-transfection, the culture medium was replaced with Dulbecco’s modified Eagle’s medium (DMEM) (Wako, Tokyo, Japan) supplemented with 10% fetal bovine serum (Gibco, Thermo Fisher Scientific). After 48 h, the culture medium was centrifuged at 1000 × *g* for 3 min to remove the cells. The resulting lentivirus-containing supernatant was used to infect RBL-2H3 mast cells in the presence of 8 μg/μL polybrene (Nacalai Tesque). Twenty-four hours post-transduction, the cells were treated with 4 μg/μL puromycin (Wako) for 2 days and then with 1 μg/μL puromycin for 8 days.

### Kinase assays and IC_50_ determination

The experiment was performed according to the method described by Kitagawa et al. (2013) and was conducted by Carna Biosciences, Inc. (Hyogo, Japan). In brief, reaction mixtures of indicated concentrations of KIRA6, 1 mM ATP, and each kinase in the presence of its substrate peptide were incubated at room temperature for 1 h (Btk, Fyn, JAK2, Kit, Lyn, Syk, Erk1, JNK1, p38α, PKCα, and PKCε) or 5 h (JAK1). Following the incubation, substrate peptides and phosphorylated peptides in the reaction mixtures were separated and quantified.

### Docking simulation

The 3D structure of human Lyn (hLyn) registered in the Protein Data Bank (PDB) contains missing coordinates. Therefore, a complete model was obtained by filling in the missing regions using AlphaFold 3 (Abramson et al., 2024). The complex structure of hLyn and KIRA6, as well as compounds with a similar scaffold to KIRA6, has not been registered in the PDB. However, a complex structure of mouse Lyn (mLyn), which has approximately 97% amino acid sequence identity with hLyn, and KIRA6, which has a similar geometric structure to PP2, has been registered. Therefore, the hLyn/KIRA6 complex structure was modeled using the mLyn/PP2 complex structure (PDBid: 2zv9). In the mLyn/PP2 complex structure, the pairs of the amino moiety of PP2 and the O atom of Glu320, the N atom of the pyrimidine ring, and the N atom of Met-322 form hydrogen bonds. From the correspondence between the partial structures of PP2 and KIRA6, it can be suggested that the amino moiety of KIRA6 and the O atom of the main chain of Glu320, along with the N atom of the pyrazine ring and the N atom of Met-322, form a hydrogen bond.

To create the hLyn/KIRA6 complex model, first, the structure of the mLyn/PP2 complex was superimposed onto the hLyn structure, and the PP2 structure was extracted. For the extracted PP2 structure, the amino moiety and the pyrazine ring of KIRA6 were superimposed onto the amino moiety and the pyrimidine ring of PP2, respectively. The superimposed structures of KIRA6 and hLyn were used as the initial structure for the hLyn/KIRA6 complex. The obtained hLyn/KIRA6 complex structure was unstable since some atomic distances between hLyn and KIRA6 were significantly close. Molecular dynamics (MD) simulations were performed in the hydration state to obtain a stable structure. As the initial structure for the MD simulation, the hLyn/KIRA6 complex structure was placed in a box of water molecules that sufficiently surrounded the complex. The parameters of the water molecule were set using the TIP3P water model. Simulations were performed on the initial structure using 2-fs timesteps, at 300 K and 1 atm. In this simulation, a 10-ns MD simulation with constraints on the interatomic distances for the two hydrogen bonds mentioned above was performed, followed by a 10-ns MD simulation without constraints. All MD simulations were performed using the AMBER software package (Götz et al., 2012; Salomon-Ferrer et al., 2013; Case et al., 2023). Electrostatic potential on hLyn was calculated using an adaptive Poisson–Boltzmann solver (APBS) (Jurrus et al., 2018).

From the structures obtained through the simulation, we extracted those in which both of the following atom pairs—(1) the amino moiety and the O atom of Glu320 and (2) the N atom of the pyrazine ring and the N atom of Met-322—are within a distance range of 2.7–3.4 Å as these pairs are suggested to be involved in hydrogen bonding between hLyn and KIRA6. The structure with the lowest system energy was selected as the solution among these.

### Western blotting

Western blotting was performed using two different methods: wet and semi-dry transfer. For wet transfer, the cells were washed with ice-cold phosphate-buffered saline (PBS) and lysed with a buffer containing 10 mM HEPES–NaOH (pH 7.5), 150 mM NaCl, 1 mM EGTA, 1 mM Na_3_VO_4_, 10 mM NaF, 10 μg/mL aprotinin, 10 μg/mL leupeptin, 1 mM phenylmethylsulfonyl fluoride (PMSF), and 1% NP-40 for 20 min. The lysates were centrifuged at 15,000 rpm for 20 min at 4 °C, and the supernatants were collected. The samples were boiled with Laemmli buffer containing 2% sodium dodecyl sulfate (SDS), 10% glycerol, 62.5 mM Tris-HCl, 5% β-mercaptoethanol, and 0.01% bromophenol blue for 3 min, fractionated using sodium dodecyl sulfate–polyacrylamide gel electrophoresis (SDS-PAGE), and transferred to nitrocellulose membranes (Wako) at 4 °C. These membranes were incubated with anti-IRE1α (#3294; Cell Signaling, Dunbarton, MA, United States) and anti-GAPDH (60004-1; Proteintech, Rosemont, IL, United States) antibodies, followed by incubation with anti-horseradish peroxidase-conjugated antibodies (MBL, Tokyo, Japan). Peroxidase binding was detected via chemiluminescence using an enhanced chemiluminescence system (Thermo Fisher Scientific). For semi-dry transfer, the samples were subjected to Mini-Protean TGX Gels (BIO-RAD, Hercules, CA, United States) and transferred to a polyvinylidene fluoride membrane, as previously described (Hiragun et al., 2013). Immunoblotting was performed using anti-Syk (#2712; Cell Signaling) and anti-phospho Syk (Tyr525/526; #2710; Cell Signaling) antibodies at 4 °C overnight. Horseradish peroxidase-conjugated secondary antibodies and chemiluminescence were used for visualization. All images were captured using a chemiluminescence detector (Fusion SOLO; VILBER, Paris, France). Finally, the density of each band was measured using ImageJ 1.37v software.

### Cell viability test

Cell viability was assessed using the cell counting kit-8 (CCK-8) (Dojindo, Kumamoto, Japan) and Cytotoxicity LDH Assay Kit-WST (Dojindo), according to the manufacturers’ protocols. Then, absorbance was measured using Varioskan LUX (Thermo Fisher Scientific).

### Measurement of intracellular Ca^2+^ concentration

Intracellular Ca^2+^ concentration was determined using the Calcium Kit-Fura 2 (Dojindo), according to the manufacturer’s protocol. Absorbance was measured every 10 s or 20 s using Varioskan Flash (Thermo Fisher Scientific). Sixty seconds after the start of measurement, BMMCs or RBL-2H3 mast cells were stimulated with DNP–BSA (50 ng/mL).

### Passive cutaneous anaphylaxis test

A mixture of 20% sulfobutylether β-cyclodextrin (Selleck Biotech, Kanagawa, Japan) and 80% saline was used to dissolve anti-DNP IgE antibodies (500 μg/mL) and KIRA6 (0.5 mg/mL), following which 100 μL was injected into the ear pinna of each 8–9-week-old BALB/c male mouse. After 24 h, 100 μL of KIRA6 (0.5 mg/mL) dissolved in 20% sulfobutylether β-cyclodextrin and 80% saline was injected into the ear pinna. After 2 h, 100 μL of the DNP–BSA solution (2 mg/mL in 10 mg/mL Evans blue solution) was intravenously administered into the tail. The mice were euthanized after 1 h. The ear was excised and soaked in 0.2 mL of 1 N KOH at 37 °C for 16 h. Then, 1.8 mL of a mixed solution of 0.6 N phosphoric acid and acetone (5:13) was added, shaken vigorously for a few seconds, and centrifuged at 2,500 rpm for 10 min. Finally, absorbance of the supernatant was measured at 620 nm using Varioskan Flash (Katayama et al., 1978; Gupta et al., 2021). All animal experiments were conducted in accordance with the NIH Guide for Care and Use of Laboratory Animals and approved by the Animal Care and Use Committee at Hiroshima University.

### Statistical analyses

The results of experiments are represented as the mean ± standard error (SE). Analysis of variance (ANOVA), followed by Dunnett’s test, was used to compare multiple groups. Statistical significance was set at *p* < 0.05 for all tests, with significance levels indicated as *p < 0.05, **p < 0.01, and ***p < 0.001 in the figure legends.

## Results

### Antigen stimulation activates IRE1α–XBP1 signaling, which is inhibited by KIRA6 in mast cells

**FIGURE 1:**
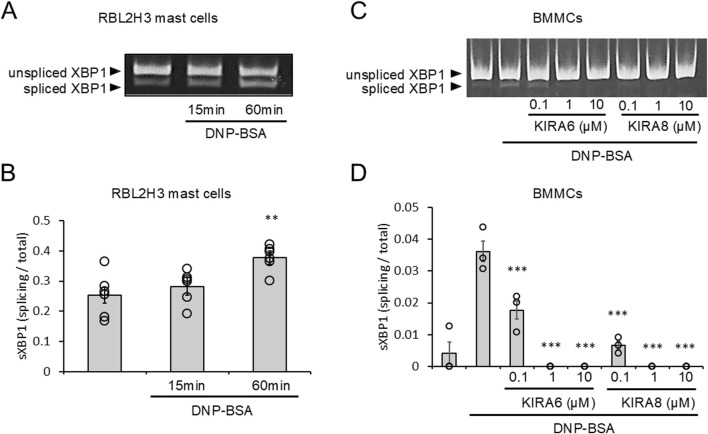
Antigen-stimulation activates IRE1α-spliced form of X-box binding protein 1 (sXBP1) signaling, which is inhibited by KIRA6. **(A)** RBL-2H3 mast cells sensitized with anti-dinitrophenyl (DNP) IgE antibodies (500 ng/mL) overnight were stimulated with antigens [20 ng/mL DNP–bovine serum albumin (BSA)] for 15 or 60 min at 37 °C (n = 6). **(B)** Densitometry of XBP1 mRNA levels in RBL-2H3 mast cells was performed using ImageJ software. **(C)** BMMCs sensitized with anti-DNP IgE antibodies (100 ng/mL) overnight were treated with IRE1α inhibitors (KIRA6 and KIRA8) at concentrations of 0.1, 1, and 10 μM for 30 min and stimulated with antigens (50 ng/mL DNP-BSA) for 30 min at 37 °C (n = 3). **(D)** Densitometry of XBP1 mRNA levels in BMMCs was performed using ImageJ software. Results are represented as the mean ± standard error (SE). **p < 0.01 and ***p < 0.001 via Dunnett’s test.

We investigated whether the IRE1α–sXBP1 axis is activated in RBL-2H3 mast cells and BMMCs in response to antigen (DNP–BSA) stimulation that induces the release of Ca^2+^ from the ER. Upon sensitization of RBL-2H3 mast cells with anti-DNP IgE antibodies and stimulation with antigens, sXBP1 levels were significantly increased within 60 min (Figures 1A,B). This suggests that antigen-activated Ca^2+^ release from the ER induces the activation of the IRE1α–sXBP1 axis. We further investigated the effects of specific IRE1α kinase inhibitors (KIRA6 and KIRA8) on the IRE1α–sXBP1 axis induced by antigen stimulation in BMMCs sensitized with anti-DNP IgE antibodies. As shown in Figures 1C,D, the induction of sXBP1 in antigen-stimulated BMMCs was significantly inhibited by KIRA6 or KIRA8. We also detected strong and rapid activation of the IRE1α–sXBP1 axis in response to the ER Ca^2+^ ATPase inhibitor, Tg, which rapidly induced Ca^2+^ release from the ER (Supplementary Figure S1). These results suggest that the IRE1α–sXBP1 axis is activated within 60 min during antigen-induced mast cell activation.

### The IRE1α inhibitor KIRA6, but not KIRA8, inhibits basophil and mast cell degranulation

**FIGURE 2:**
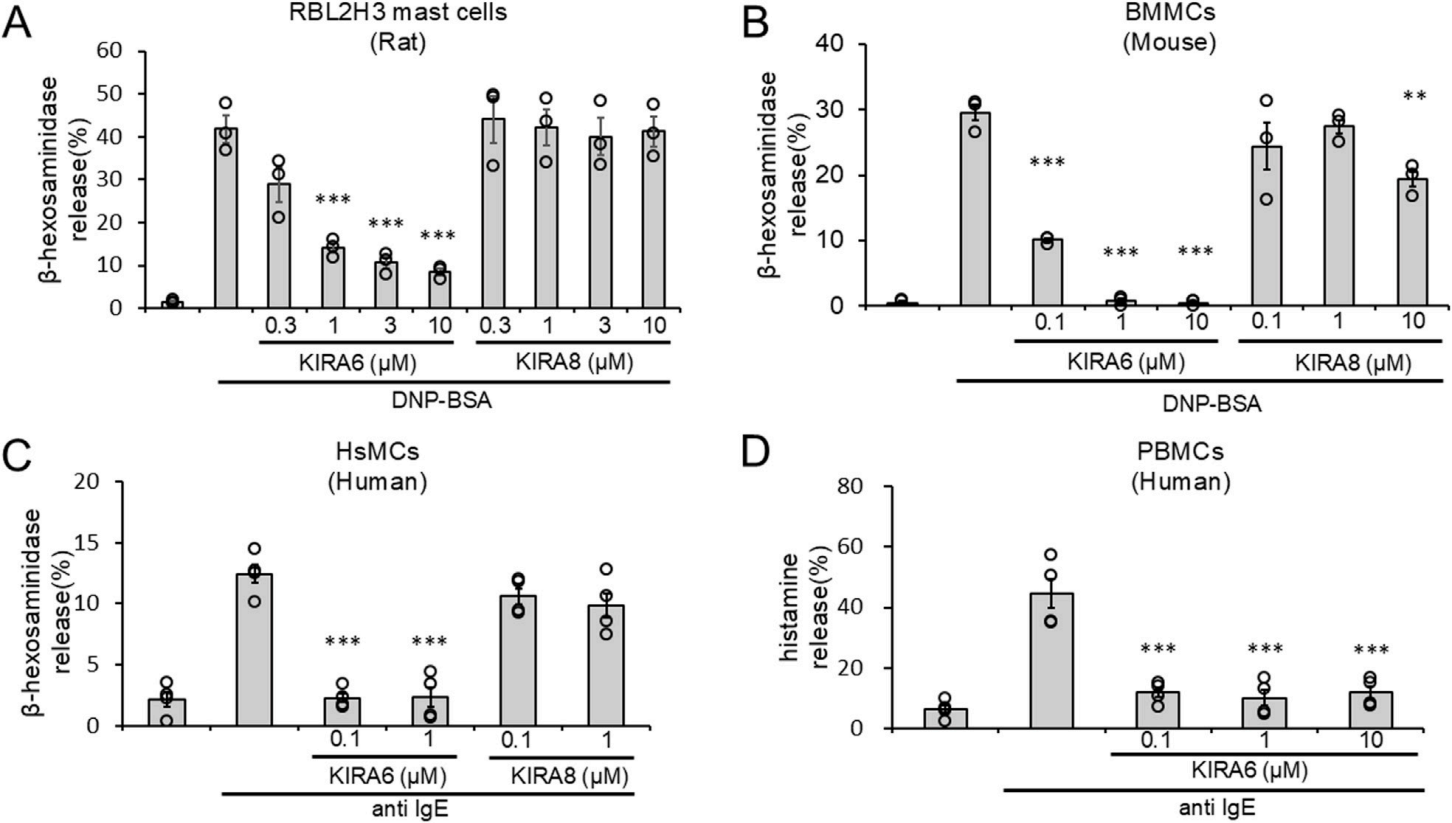
The IRE1α inhibitor KIRA6, but not KIRA8, inhibits basophil and mast cell degranulation. **(A)** RBL-2H3 mast cells (rat) sensitized with anti-DNP IgE antibodies (500 ng/mL) overnight were treated with IRE1α inhibitors (KIRA6 and KIRA8) at the indicated concentrations for 30 min and stimulated with antigens (100 ng/mL DNP-BSA) for 15 min at 37 °C (n = 3). **(B)** BMMCs (mouse) sensitized with anti-DNP IgE antibodies (100 ng/mL) overnight were treated with IRE1α inhibitors (KIRA6 and KIRA8) for 30 min at the indicated concentrations and stimulated with antigens (50 ng/mL DNP-BSA) for 15 min at 37 °C (n = 3). **(C)** Human skin mast cells (hsMCs) sensitized with human IgE antibodies (50 ng/mL) overnight were treated with the IRE1α inhibitor KIRA6 for 30 min and stimulated with anti-IgE antibodies (670 ng/mL) for 15 min at 37 °C (n = 4). **(D)** Human PBMCs, including basophils, from peripheral blood were treated with the IRE1α inhibitor KIRA6 for 30 min and stimulated with anti-IgE antibodies (670 ng/mL) for 15 min at 37 °C (n = 4). Results are represented as the mean ± SE. **p < 0.01 and ***p < 0.001 via Dunnett’s test.

Next, we investigated the effects of KIRA6 and KIRA8 on basophil and mast cell degranulation. We examined the effects of KIRA6 and KIRA8 on the antigen-induced degranulation of RBL-2H3 mast cells and BMMCs sensitized with anti-DNP IgE antibodies. Interestingly, as shown in Figures 2A,B, KIRA6, but not KIRA8, significantly inhibited the degranulation of RBL-2H3 mast cells and BMMCs in a concentration-dependent manner (n = 3). In contrast, PERK (GSK2606414) and ATF-6 (Ceapin-A7) inhibitors did not significantly suppress BMMC degranulation (n = 3) (Supplementary Figure S2). We further investigated whether KIRA6 suppresses degranulation in human-derived mast cells and basophils. This system included hsMCs sensitized to human IgE antibodies and PBMCs, including basophils, in response to anti-IgE antibodies. As shown in Figures 2C,D, KIRA6 significantly inhibited the IgE receptor-associated activation of hsMCs and human basophils (n = 4). KIRA6 did not affect cell viability (n = 3) (Supplementary Figures S3A, S3B). These results highlight KIRA6 as a unique compound inhibiting mast cell- and basophil-induced allergic reactions in response to antigens, without any cytotoxicity.

### KIRA6 inhibits the production and release of lipid mediators and pro-inflammatory cytokines by mast cells in response to antigen stimulation

**FIGURE 3:**
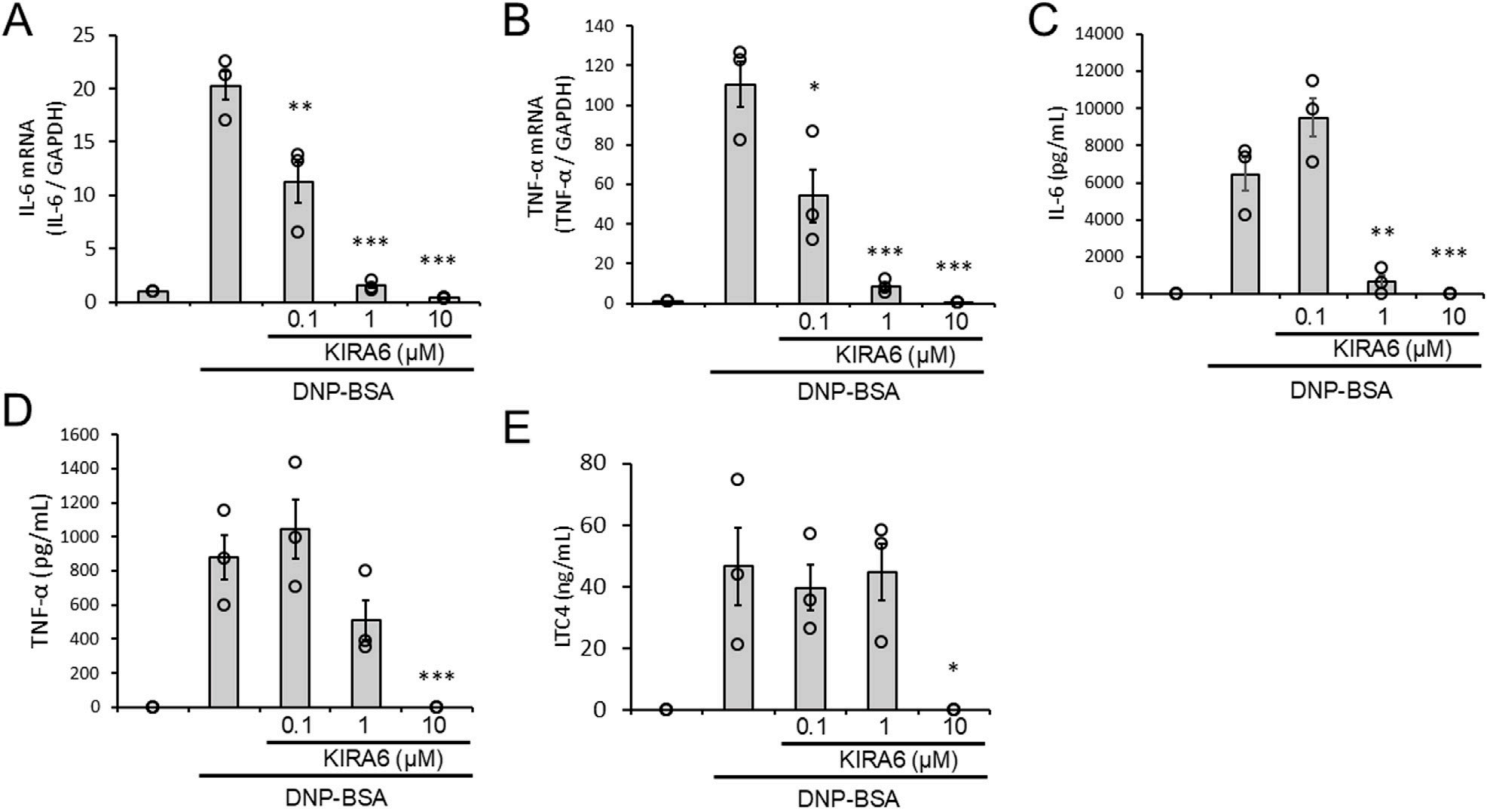
KIRA6 inhibits the production and release of lipid mediators and pro-inflammatory cytokines by mast cells in response to antigen stimulation. **(A,B)** BMMCs sensitized with anti-DNP IgE antibodies (100 ng/mL) overnight were treated with the IRE1α inhibitor KIRA6 for 30 min at the indicated concentrations and stimulated with antigens (50 ng/mL DNP-BSA) for 30 min at 37 °C (n = 3). **(C–E)** BMMCs sensitized with anti-DNP IgE antibodies (100 ng/mL) overnight were treated with the IRE1α inhibitor KIRA6 for 30 min at the indicated concentrations and stimulated with antigens (50 ng/mL DNP-BSA) for 24 h (IL-6), 3 h (TNF-α), or 1 h (LTC4) at 37 °C (n = 3). Results are represented as the mean ± SE. *p < 0.05, **p < 0.01, and ***p < 0.001 via Dunnett’s test.

We analyzed whether KIRA6 inhibits the production of IL-6 and TNFα mRNA and the release of IL-6, TNFα, and LTC4 by BMMCs in response to antigen stimulation. KIRA6 significantly inhibited antigen-induced IL-6 and TNFα mRNA induction in BMMCs sensitized with anti-DNP IgE antibodies (n = 3) (Figures 3A,B). Additionally, KIRA6 inhibited the antigen-induced release of IL-6, TNFα, and LTC4 from BMMCs sensitized with anti-DNP IgE antibodies (n = 3) (Figures 3C–E). These results suggest that KIRA6 suppresses the aggravation of allergic reactions by inhibiting the production and release of lipid mediators and pro-inflammatory cytokines.

### IRE1α depletion does not affect the antigen-induced activation of mast cells

**FIGURE 4:**
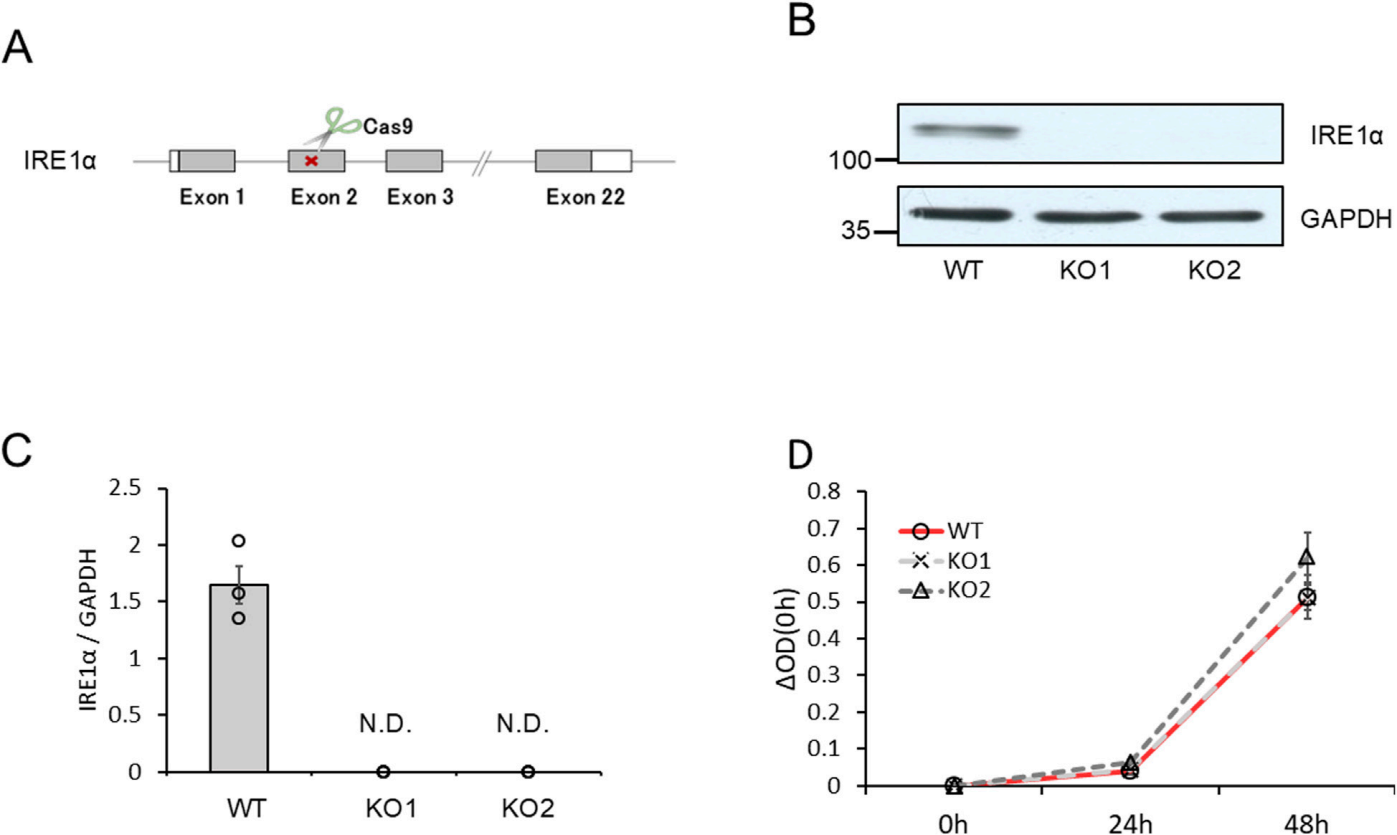
IRE1α-knockout RBL-2H3 mast cells (KO1 and KO2) were developed using the CRISPR/Cas9 system. **(A)** Schematic representation of IRE1α-knocked-out RBL-2H3 mast cell generation using CRISPR/Cas9 system. **(B)** Protein expression levels of IRE1α and GAPDH in wild-type (WT), KO1, and KO2 cells determined via Western blotting (semi-dry; n = 3). **(C)** Densitometric analysis of IRE1α and GAPDH levels using ImageJ image analysis software. **(D)** Survival and proliferation rates of WT, KO1, and KO2 cells measured using the cell counting kit-8 (n = 3). Results are represented as the mean ± SE. N.D., not detected.

**FIGURE 5:**
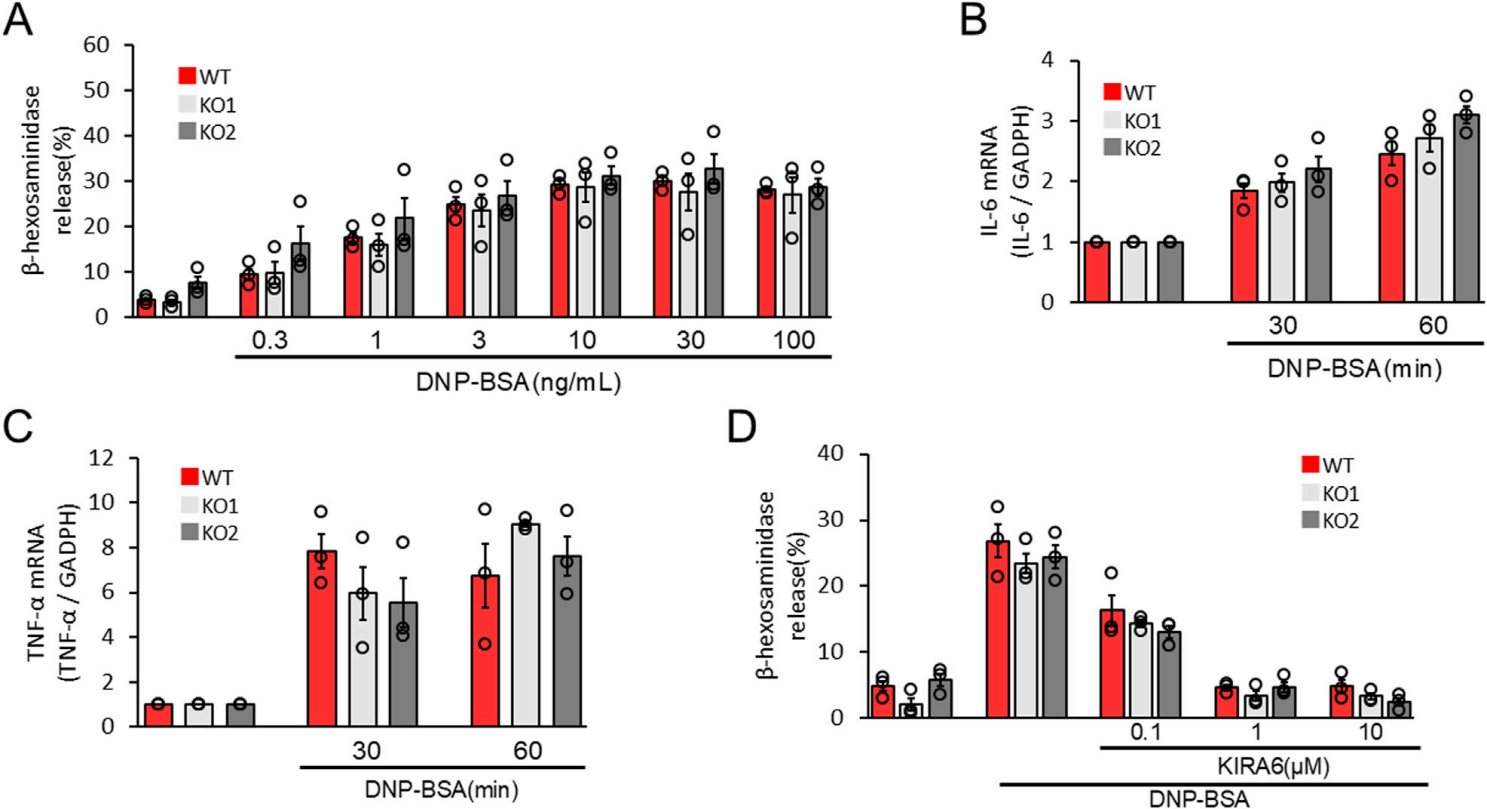
IRE1α deficiency does not affect antigen-induced RBL-2H3 mast cell activation. **(A)** WT, KO1, and KO2 cells sensitized with anti-DNP IgE antibodies (100 ng/mL) overnight were stimulated with antigens (DNP–BSA) at the indicated concentrations for 15 min at 37 °C (n = 3). **(B,C)** WT, KO1, and KO2 cells sensitized with anti-DNP IgE antibodies (100 ng/mL) overnight were stimulated with antigens (50 ng/mL DNP–BSA) for 30 and 60 min at 37 °C (n = 3). **(D)** WT, KO1, and KO2 cells sensitized with anti-DNP IgE antibodies (100 ng/mL) overnight were treated with the IRE1α inhibitor KIRA6 at the indicated concentrations for 30 min and stimulated with antigens (50 ng/mL DNP-BSA) for 15 min at 37 °C (n = 3). Results are represented as the mean ± SE.

To confirm the roles of the IRE1α–sXBP1 axis in the functions of mast cells on allergic responses, we developed two IRE1α-knocked-out (KO) RBL-2H3 mast cells, KO1 and KO2. We used the clustered regularly interspaced palindromic repeat (CRISPR)/CRISPR-associated protein 9 (Cas9) system to delete the coding region of IRE1α (Figure 4A). Figures 4B,C show the expression levels of IRE1α in wild-type (WT), KO1, and KO2 RBL-2H3 cells determined via Western blotting. Protein expression of IRE1α was not detected in KO1 and KO2 cells. Notably, depletion of IRE1α did not affect the survival and proliferation of RBL-2H3 mast cells (Figure 4D). Then, we performed degranulation assays using WT, KO1, and KO2 cells in response to antigen stimulation. Unexpectedly, antigen-induced degranulation of KO1 and KO2 cells was not altered compared to that of WT cells (Figure 5A). Moreover, antigen-induced production of IL-6 and TNFα mRNA in KO1 and KO2 cells was comparable to that in WT cells (Figures 5B,C). KIRA6 treatment at 1 μM significantly suppressed the antigen-induced degranulation of KO1 and KO2 cells by more than 90% (Figure 5D). KIRA6 also suppressed the antigen-induced increase in intracellular Ca^2+^ concentrations in IRE1α KO cells (Supplementary Figure S4). These results suggest that KIRA6 inhibits antigen-induced mast cell activation via an IRE1α-independent pathway.

### KIRA6 inhibits antigen-induced tyrosine kinase activation and intercellular Ca^2+^ mobilization in mast cells

**FIGURE 6:**
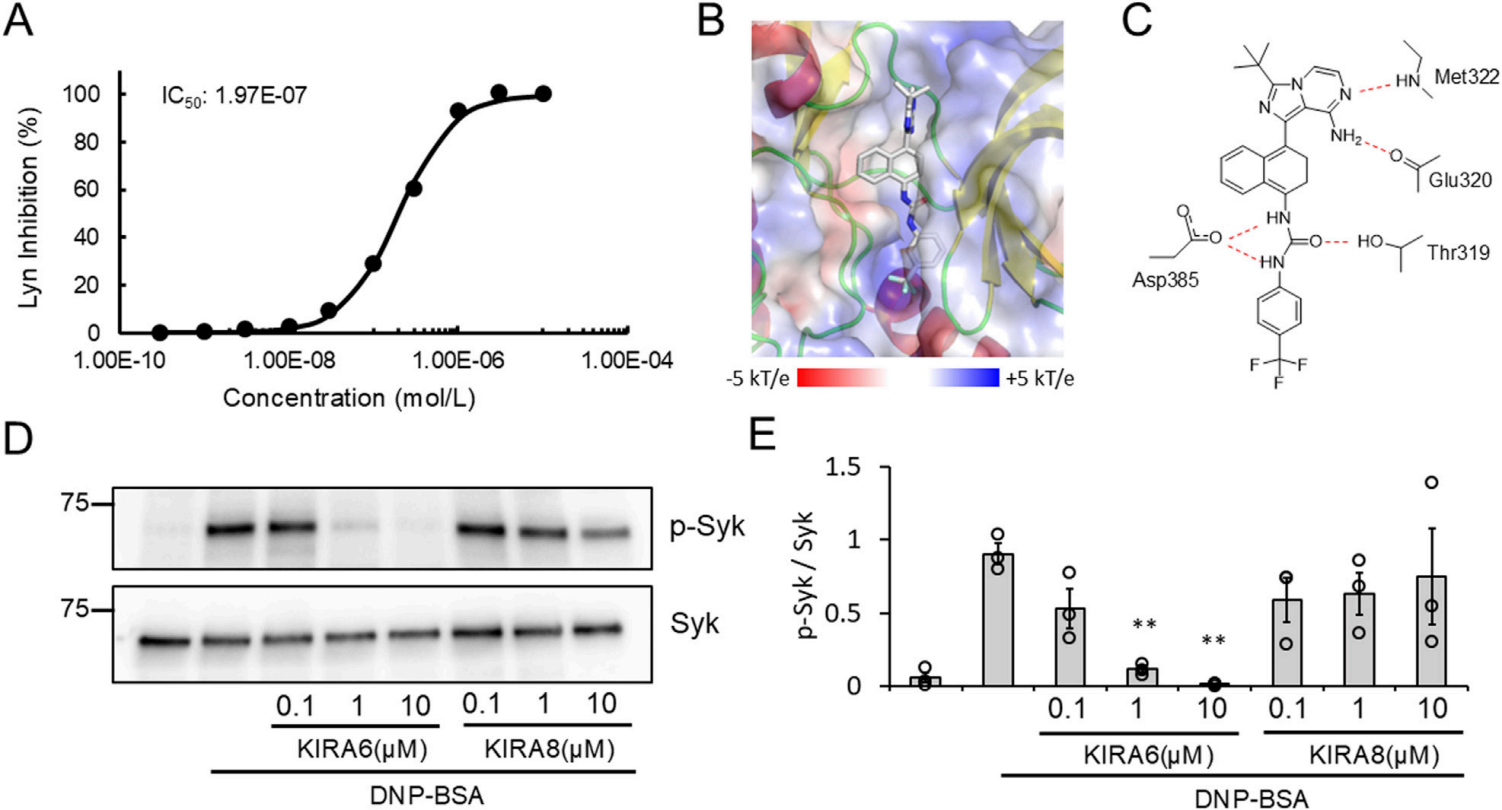
KIRA6 inhibits antigen-induced tyrosine kinase activation in mast cells. **(A)** The dose-dependent inhibition curve of KIRA6 on kinase activity of Lyn was determined using KIRA6 at concentrations ranging from 0.0003 to 10 μM, based on a duplicate assay. **(B)** The position of KIRA6 in the hLyn/KIRA6 complex is shown. The structures of hLyn and KIRA6 are represented using the cartoon model and stick model (C: white, N: blue, O: red, and F: cyan), respectively. Electrostatic potential shown ranging from −5 kT/e (red) to +5 kT/e (blue) on hLyn is mapped onto a surface model. **(C)** A schematic diagram of the hydrogen binding sites between hLyn and KIRA6 is presented. **(D)** BMMCs sensitized with anti-DNP IgE antibodies (100 ng/mL) overnight were treated with IRE1α inhibitors (KIRA6 and KIRA8) at the indicated concentrations for 30 min and stimulated with antigens (50 ng/mL DNP-BSA) for 1 min at 37 °C (n = 3). Protein expression levels of spleen-associated tyrosine kinase (Syk) and p-Syk in BMMCs were determined via Western blotting (wet). **(E)** Densitometric analysis of Syk and p-Syk protein levels was performed using ImageJ image analysis software. Results are represented as the mean ± SE. **p < 0.01 via Dunnett’s test.

As described above, KIRA6 affected mast cell degranulation via the IRE1α–sXBP1-independent pathway (Figure 5D). Then, what is the target molecule of KIRA6 in inhibiting allergic reactions? To explore other pharmacological targets of KIRA6 in mast cells and basophils, we then examined the effects of KIRA6 on several tyrosine kinases and serine/threonine kinases. The assay was performed by Carna Biosciences, Inc. (Hyogo, Japan). As shown in Table 1, KIRA6 mainly inhibited the src family tyrosine kinases, such as Fyn and Lyn. The dose-dependent inhibition curve of KIRA6 on Lyn is shown in Figure 6A. We then investigated whether KIRA6 can directly bind to hLyn using *in silico* binding simulation. In the hLyn/KIRA6 complex model, KIRA6 stably binds to the ATP-binding site by forming five hydrogen bonds with amino acid residues of hLyn (Figures 6B,C). In addition to the two hydrogen bonds used to evaluate the model (the amino group of KIRA6 and the oxygen atom of the main chain of Glu320, along with the N atom of the pyrazine ring and the NH of Met-322), the carboxyl group of Asp385 is bonded to the two NHs of the urea group, and the OH of Thr319 is hydrogen-bonded to the O atom. In general, hydrogen bonds strengthen compound–protein interactions, suggesting that KIRA6 binds to hLyn with high affinity. These hydrogen bonds, therefore, contribute to the lowest energy among the 15 structures. These findings indicate that the complex may adopt a structure similar to the proposed model. Lyn plays an important role in antigen-induced activation, which phosphorylates its downstream target, such as Syk (Gilfillan and Tkaczyk, 2006; Siraganian et al., 2010). Furthermore, Syk activates various molecules, including Bruton’s tyrosine kinase (Btk), thereby facilitating the transmission of signals downstream (Gilfillan and Tkaczyk, 2006; Siraganian et al., 2010). Therefore, in the present study, we focused on the downstream events of Lyn, such as Syk phosphorylation and Ca^2+^ mobilization. To this end, we investigated the effect of KIRA6 on antigen-induced phosphorylation of Syk in BMMCs sensitized with anti-DNP IgE antibodies and found that KIRA6 at 1 μM inhibited Syk phosphorylation by more than 90% (Figures 6D,E). In addition, KIRA6 inhibited antigen-induced Ca^2+^ mobilization in BMMCs sensitized with anti-DNP IgE antibodies (Supplementary Figure S5). These results suggest that KIRA6 binds to Lyn and blocks the activation of Syk by inhibiting the kinase activity of Lyn, thereby suppressing mast cell and basophil degranulation.

**TABLE 1:** Inhibition of kinase activities by KIRA6. Reaction mixtures containing the kinase, its substrate, and KIRA6 (0.1 or 1 μM) were incubated, and substrate phosphorylation was assessed. The assay was performed by Carna Biosciences, Inc. (Hyogo, Japan).

Type of kinase	Kinase	Inhibition (%)	
		KIRA6 (μM)	
		0.1	1
Tyrosine kinase	Btk	46.2	91.7
	Fyn	14.9	81.4
	JAK1	1.1	7.9
	JAK2	0.1	3.2
	KIT	6.3	82.3
	Lyn	38.9	94.2
	Syk	−1.4	0.5
Serine/threonine kinase	Erk1	−0.6	5.5
	JNK1	2.1	4.1
	p38α	49.4	96.0
	PKCα	1.5	0.5
	PKCε	6.9	1.6

### KIRA6 suppresses antigen-induced basophil and mast cell degranulation more potently than clinically used anti-allergic drugs

**FIGURE 7:**
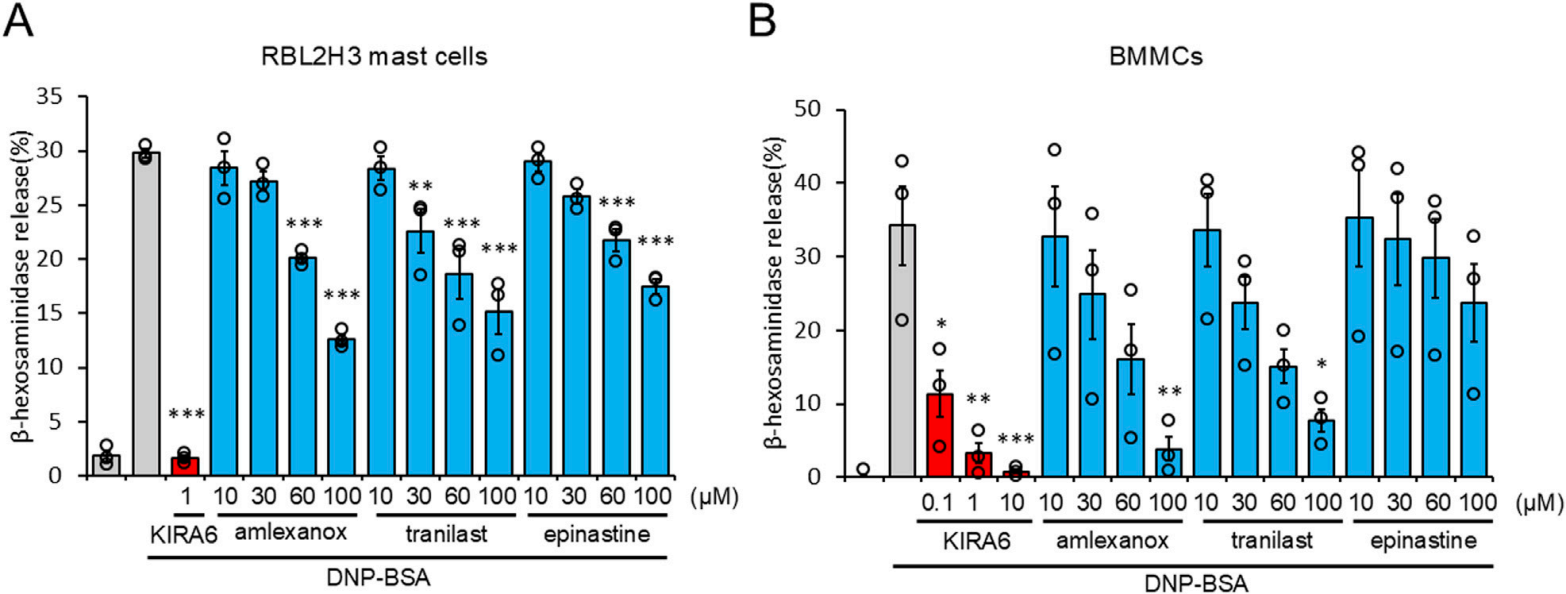
KIRA6 suppresses antigen-induced basophil and mast cell degranulation more potently than clinically used anti-allergic drugs. **(A)** RBL-2H3 mast cells sensitized with anti-DNP IgE antibodies (500 ng/mL) overnight were treated with inhibitors (KIRA6, amlexanox, tranilast, and epinastine) at the indicated concentrations for 30 min and stimulated with antigens (100 ng/mL DNP-BSA) for 15 min at 37 °C (n = 3). **(B)** BMMCs sensitized with anti-DNP IgE antibodies (100 ng/mL) overnight were treated with inhibitors (KIRA6, amlexanox, tranilast, and epinastine) at the indicated concentrations of 0.1, 1, and 10 μM for KIRA6 and at 10, 30, 60, and 100 μM for amlexanox, tranilast, and epinastine, respectively, for 30 min, and then stimulated with antigens (50 ng/mL DNP-BSA) for 15 min at 37 °C (n = 3). Results are represented as the mean ± SE. *p < 0.05, **p < 0.01, and ***p < 0.001 via Dunnett’s test.

As KIRA6 exerts anti-allergic effects by inhibiting degranulation, we compared its anti-allergic effects with those of the clinically used drugs, such as amlexanox, tranilast, and epinastine. They are selected as benchmark drugs because they are widely used and known to inhibit the release of chemical mediators such as histamine from mast cells and basophils (Makino et al., 1987; Komatsu et al., 1988; Fujishima et al., 2014). As shown in Figures 7A,B, KIRA6 markedly inhibited the antigen-induced degranulation of RBL-2H3 mast cells and BMMCs sensitized with anti-DNP IgE antibodies at extremely low concentrations (over 100 times lower concentrations) compared to the known compounds used in clinical practice (amlexanox, tranilast, and epinastine). Overall, low concentrations (<1 μM) of KIRA6 effectively suppressed antigen-induced allergic reactions better than existing anti-allergic drugs, without any cytotoxicity (Supplementary Figures S3A, S3B).

### KIRA6 inhibits antigen-induced hyperpermeability *in vivo*

**FIGURE 8:**
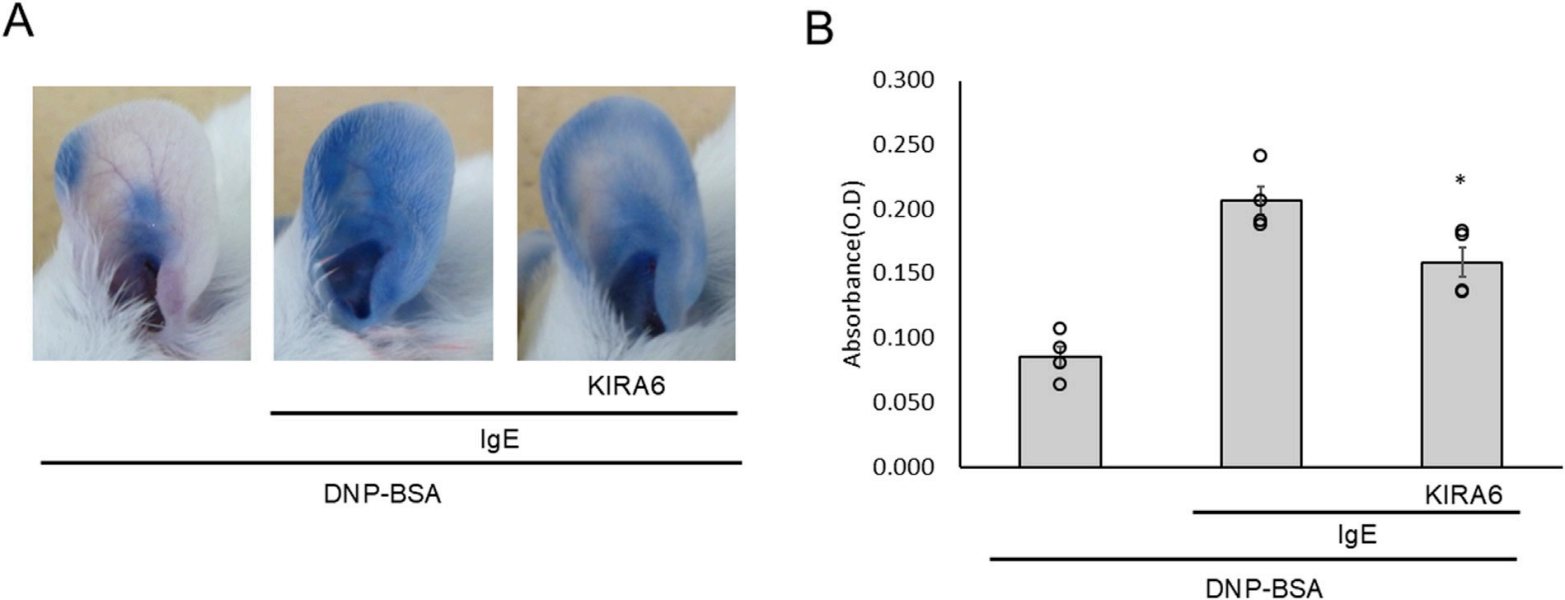
KIRA6 inhibits antigen-induced hyperpermeability *in vivo*. **(A)** A mixture of 20% sulfobutylether β-cyclodextrin (SBE-β-CD) and 80% saline was used to dissolve DNP–IgE antibodies (500 μg/mL) and KIRA6 (0.5 mg/mL), and 100 μL was injected into the ear pinna of each mouse. After 24 h, 100 μL of KIRA6 (0.5 mg/mL) dissolved in 20% SBE-β-CD and 80% saline was again injected into the ear pinna. After 2 h, 100 μL of the DNP–BSA solution (2 mg/mL in 10 mg/mL Evans blue solution) was intravenously (i.v.) administered into the tail vein. The mice were euthanized after 1 h, and photographs of their ears were taken. **(B)** Evans blue dye was extracted from the ears as described in the *Methods* section, then centrifuged, and measured for absorbance at 620 nm (n = 4).Results are represented as the mean ± SE. *p < 0.05 via Dunnett’s test.

To further investigate the anti-allergic effects of KIRA6 *in vivo*, we conducted a passive cutaneous anaphylaxis (PCA) test in BALB/c mice. PCA is a well-established test for local allergic reactions induced by injecting anti-DNP IgE antibodies into the mouse ear and antigens (DNP-BSA) into the tail vein (Nam et al., 2017). Intradermal administration of KIRA6 into the ear significantly reduced antigen-induced hyperpermeability (Figures 8A,B). These results confirmed that KIRA6 exerts potent anti-allergic effects *in vivo*.

## Discussion

In this study, we evaluated the roles of ER stress-related molecules in mast cell activation. In RBL-2H3 mast cells and BMMCs, sXBP1 levels increased in response to antigen stimulation (Figures 1A–D). sXBP1 levels were also increased by Tg, which depleted Ca2+ in the ER and induced mast cell degranulation (Supplementary Figure S1). These results suggest the activation of the IRE1α–sXBP1 axis upon mast cell activation by antigens. Moreover, we found that KIRA6 and KIRA8, which serve as IRE1α kinase inhibitors, inhibited sXBP1 production in BMMCs (Figures 1C,D). Furthermore, KIRA6 suppressed antigen-induced degranulation of RBL-2H3 mast cells and BMMCs (Figures 2A,B). However, we found that antigen-induced degranulation was induced in IRE1α-KO RBL-2H3 mast cells (Figure 5A). Moreover, KIRA6 inhibited antigen-induced degranulation in IRE1α-KO RBL-2H3 mast cells (Figure 5D). These results suggest that KIRA6 may exert its effect through targets other than IRE1α. Although KIRA8 has higher selectivity and potency for IRE1α than KIRA6 (Morita et al., 2017), it did not suppress the degranulation of RBL-2H3 mast cells and BMMCs at all (Figures 2A,B). Additionally, inhibitors of both PERK (GSK2606414) and ATF-6 (Ceapin-A7) did not significantly inhibit the antigen-induced degranulation of BMMCs (Supplementary Figure S2). These findings suggest KIRA6 as a unique compound suppressing antigen-induced degranulation differently from other compounds. Furthermore, KIRA6 effectively suppressed degranulation even in human skin mast cells and peripheral blood basophils (IgE antibody-related degranulation of hsMCs and peripheral blood basophils; Figures 2C,D). Thus, KIRA6 has a unique property in inhibiting antigen-induced degranulation of mast cells and basophils.

To explore the anti-allergic potential of KIRA6, we further investigated its effects on the production and release of pro-inflammatory mediators by mast cells. The production and release of pro-inflammatory mediators, such as IL-6, TNF-α, and LTC4, by mast cells are closely related to the exacerbation of allergic reactions (Wang and Liu, 2024; Sasaki and Yokomizo, 2019). In this study, we found that KIRA6 suppressed the production and release of antigen-induced LTC4, IL-6, and TNF-α by BMMCs (Figures 3A–E). In contrast, KIRA6 did not affect cell death (LDH release) and survival of BMMCs (Supplementary Figures S3A, S3B), suggesting that KIRA6 significantly inhibits mast cell activation without any toxic effects. Therefore, KIRA6 shows great potential as an anti-allergic drug by inhibiting antigen-induced mast cell and basophil activation, including their degranulation and the release of pro-inflammatory cytokines.

Although the IRE1α inhibitor KIRA6 inhibited mast cell and basophil activation, the mechanisms by which IRE1α regulates antigen-induced mast cell and basophil activation remain unclear. Therefore, we established IRE1α-KO RBL-2H3 mast cell lines to investigate the roles of IRE1α in mast cell and basophil allergic functions. Unexpectedly, IRE1α-KO RBL-2H3 mast cells (KO1 and KO2) did not affect antigen-induced degranulation and IL-6 and TNF-α mRNA induction (Figures 5A–C). Although Martinon et al. (2010) reported that XBP1 in macrophages is essential for the sustained production of mediators, such as IL-6, allergic response-mediated IL-6 induction may not be associated with the IRE1α–XBP1 system. Overall, these findings suggest that cytokine production mediated by antigen stimulation is regulated independently of the IRE1α–sXBP1 pathway. KIRA6 also suppressed antigen-induced degranulation and Ca^2+^ mobilization in IRE1α-KO cells (Figure 5D; Supplementary Figure S4). Thus, IRE1α possibly does not play any role in antigen-induced mast cell and basophil activation, suggesting the presence of other target molecules of KIRA6 involved in the antigen-induced activation of these cells. Subsequently, we analyzed the possible target molecules of KIRA6.

**FIGURE 9:**
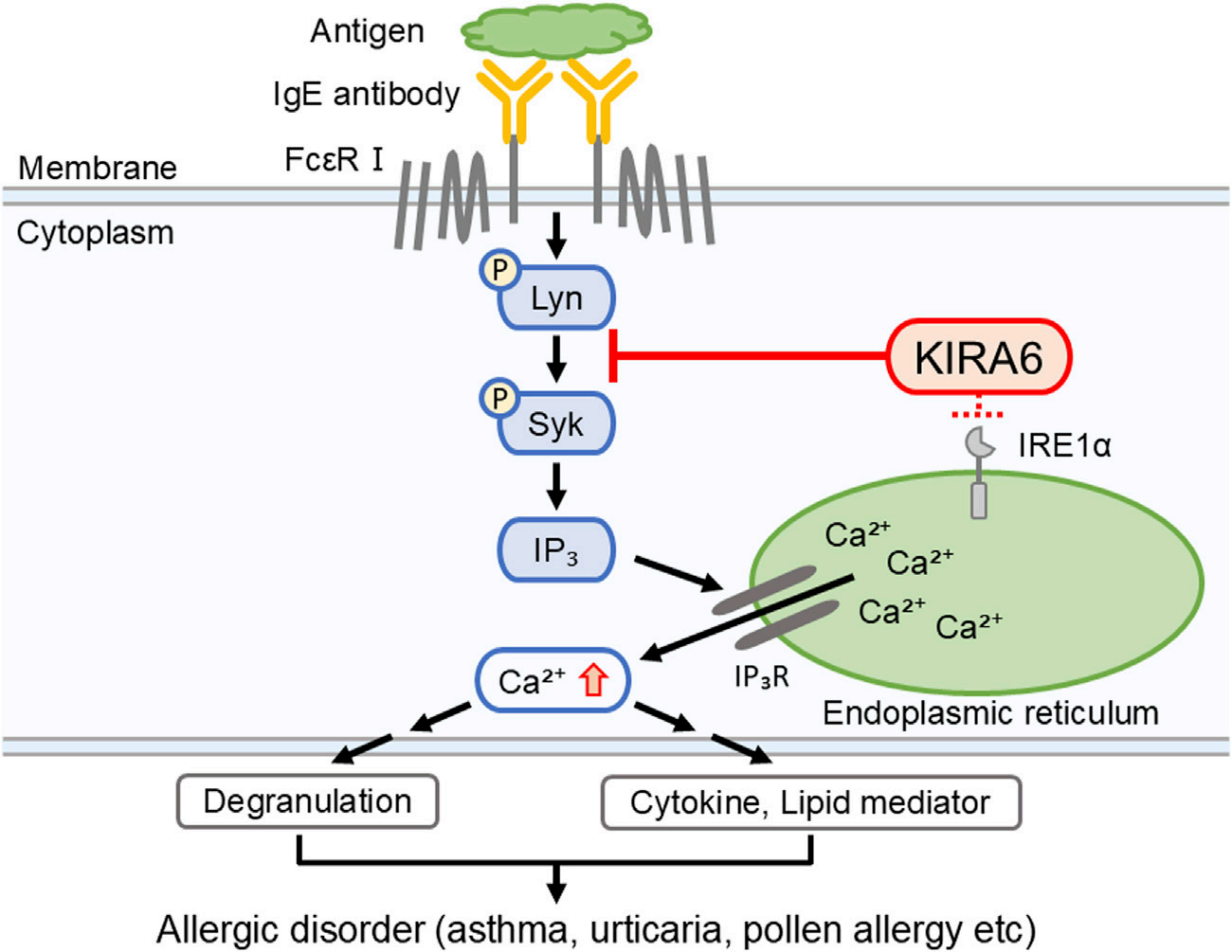
KIRA6 inhibits the activation of mast cells and basophils by suppressing the function of tyrosine kinases, although it also has inhibitory effects on IRE1α. This figure illustrates a hypothetical model describing how KIRA6 inhibits antigen-induced activation of mast cells and basophils. KIRA6 possibly blocks kinase activity of Lyn, thereby inhibiting antigen-induced allergic reactions.

Mast cells and basophils express FcεRI on their surfaces. When FcεRI is crosslinked with IgE antibodies and specific antigens, several tyrosine kinases, including Lyn and Syk, are activated, resulting in degranulation, Ca^2+^ mobilization, and the release of pro-inflammatory molecules, such as IL-6, TNF-α, and LTC4 (Gilfillan and Rivera, 2009). To identify the target molecules of KIRA6, we examined its effect on several kinases and found that it inhibited the kinase activities of Btk, Fyn, Kit, Lyn, and p38α (Table 1; Figure 6A). Moreover, we clarified that KIRA6 binds to the ATP-binding site of Lyn through binding simulation (Figures 6B,C). Lyn plays important roles in the early signaling events downstream of FcεRI activation in mast cells and contributes to the activation of Syk and Btk (Gilfillan and Tkaczyk, 2006; Siraganian et al., 2010). Fyn kinase also plays a critical role in FcεRI-mediated mast cell degranulation, independently of the Lyn pathways (Parravicini et al., 2002; Barbu et al., 2010). However, the detailed roles of Fyn in mast cell and basophil activation remain unclear. KIRA6 has been reported to inhibit the receptor tyrosine kinase KIT, a receptor of stem cell factor (Mahameed et al., 2019). However, KIT is not activated by antigen (Tkaczyk et al., 2004). Although p38 is not critical for degranulation, p38 may be involved in the production and release of lipid mediators and cytokines (Chue et al., 2004; Kalesnikoff et al., 2002). Collectively, these observations suggest that KIRA6 may inhibit antigen-induced mast cell activation through the inhibition of the kinase activities of Lyn and possibly Fyn. Recently, Wunderle et al. (2025) also reported that KIRA6 inhibited mast cell activation by binding to Lyn and Fyn. Therefore, in the present study, we focused on the downstream events of Lyn as the Lyn/Syk signaling pathway plays an extremely important role in antigen-induced degranulation (Gilfillan and Tkaczyk, 2006). To this end, we examined the effect of KIRA6 on antigen-induced activation of Syk. Notably, KIRA6 significantly inhibited the antigen-induced phosphorylation of Syk in mast cells (Figures 6D,E). These results suggest that KIRA6 inhibits the phosphorylation of Syk by suppressing the kinase activity of Lyn. Moreover, KIRA6 inhibited intracellular Ca^2+^ mobilization triggered by Syk activation (Supplementary Figure S5). On the other hand, the inhibitory effect of KIRA6 on Ca^2+^ mobilization was slightly weaker than that on Syk activation, possibly due to the complexity of antigen-induced signaling pathways (Gilfillan and Tkaczyk, 2006). Overall, these results suggest that KIRA6 suppresses the activation of antigen-induced mast cells and basophils by inhibiting the kinase activity of Lyn, followed by the activation of Syk (Figure 9). These findings indicate that the suppression of tyrosine kinases, such as Lyn, but not IRE1α, is critical for the anti-allergic effects of KIRA6. However, KIRA6 also suppresses several kinase activities in multiple cells, such as immune cells. Therefore, the effect of KIRA6 on the functions of other immune cells should be investigated in the future.

We also investigated the therapeutic impacts of KIRA6 on allergies. As KIRA6 inhibited basophil and mast cell activation at low concentrations (0.1–1 μM), we compared its effectiveness with that of existing drugs clinically used for inhibiting histamine release. Amlexanox, epinastine, and tranilast are clinically used anti-allergic drugs that inhibit mast cell activation (Makino et al., 1987; Komatsu et al., 1988; Fujishima et al., 2014). Notably, KIRA6 strongly suppressed RBL-2H3 mast cell and BMMC degranulation at lower concentrations (<1 μM) than that of the existing drugs (Figures 7A,B). These results suggest KIRA6 has potential as an anti-allergy drug.

As KIRA6 effectively inhibited allergic reactions, we further evaluated its efficacy *in vivo*. The PCA models are well-established animal models to evaluate localized immediate allergic reactions (Kim et al., 2018; Kim et al., 2017). To investigate whether KIRA6 suppresses mast cell and basophil activation *in vivo,* we performed PCA using anti-DNP IgE antibody-sensitized mice. Intradermal administration of KIRA6 significantly inhibited antigen stimulation-induced hyperpermeability (Figures 8A,B). These results suggest KIRA6 as an effective therapeutic for allergies. However, the effect of KIRA6 *in vivo* appears to be weaker than that observed *in vitro*, which may be due to its limited solubility and unknown pharmacokinetic profile. To improve its *in vivo* efficacy, further studies are needed to clarify its pharmacokinetics, including absorption, distribution, metabolism, and excretion. In addition, optimizing its formulation and physicochemical properties, such as solubility and hydrophilicity, along with exploring structural modifications and suitable solvents, may enhance its therapeutic potential. Moreover, evaluating its effects in other *in vivo* allergic models, such as anaphylaxis, rhinitis, and asthma, will be important for the development of KIRA6-based anti-allergic drugs.

In this study, we did not evaluate the toxicity of KIRA6 *in vivo*. However, KIRA6 was administered intraperitoneally twice daily for over 4 weeks in a study involving male Ins2^+/Akita^ mice (5 mg/kg) without apparent adverse effects (Ghosh et al., 2014), suggesting a certain degree of *in vivo* safety. Nevertheless, the safety profile of KIRA6 in humans remains unexplored. Therefore, future research should address these aspects in detail to support the clinical application of KIRA6-based therapies.

## Conclusion

In conclusion, we clearly demonstrated that KIRA6 significantly inhibited the activation of rodent/human mast cells and basophils *in vitro* and *in vivo* via an IRE1α-independent pathway, using multiple techniques such as kinase assays and the generation of knockout cells. Instead of IRE1α, one of the targets of KIRA6 may be the Lyn/Syk-dependent pathway, which is an important allergic pathway. Our findings suggest that KIRA6 and its structural analogs are potential therapeutic drugs for various allergic diseases.

## Data Availability

The original contributions presented in the study are included in the article/Supplementary Material; further inquiries can be directed to the corresponding authors.
