# Supplementary material for: Unique compound with anti-allergic action: inhibition of Lyn kinase activity by KIRA6
Source: Front Pharmacol. 2025 Jul 25;16:1625798. doi: 10.3389/fphar.2025.1625798 (PMC12331723; doi:10.3389/fphar.2025.1625798)
Supplement: Supplementary file 1 [file DataSheet1.pdf]

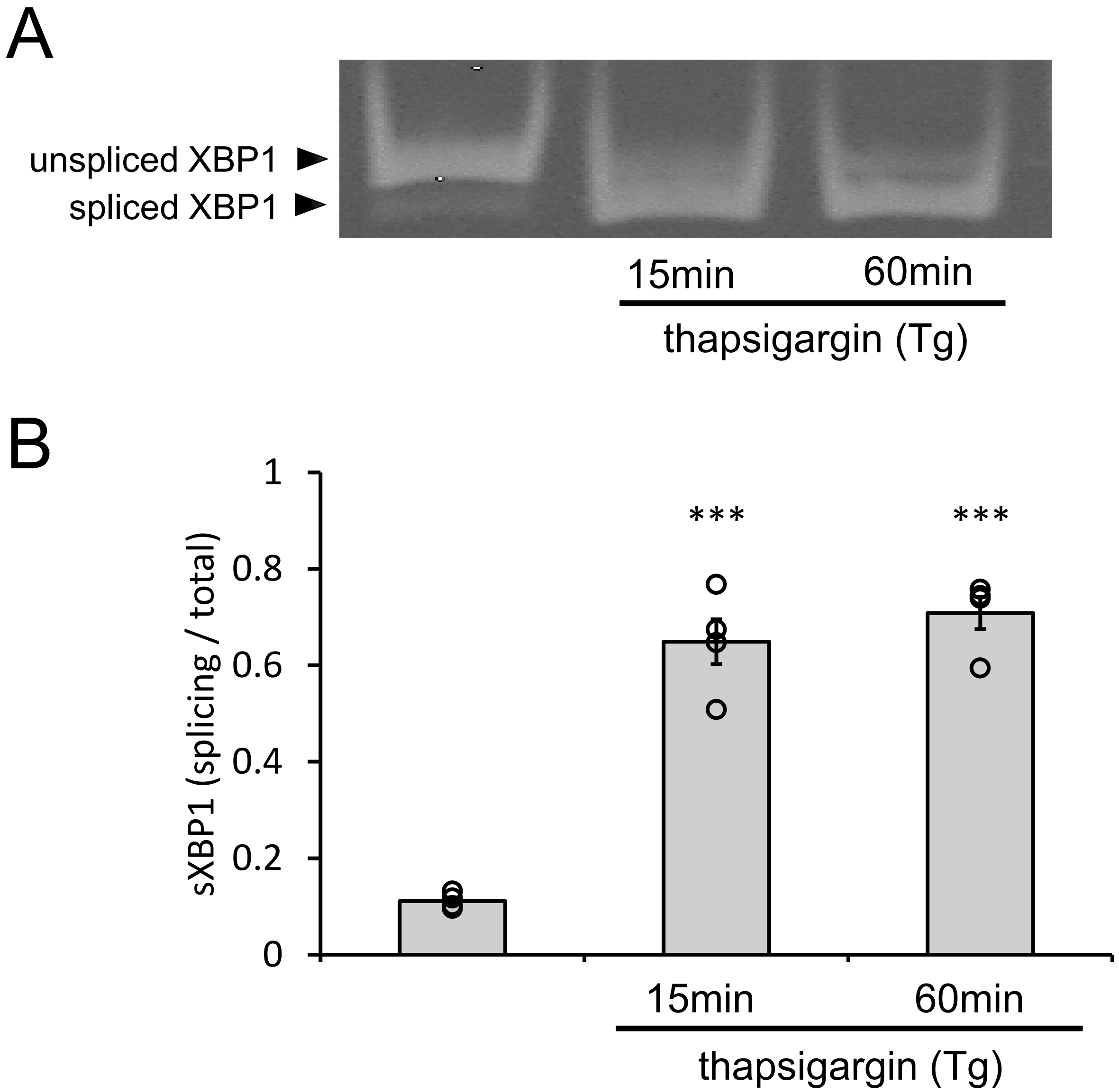

Supplementary figure 1. Thapsigargin (Tg) induces X-box-binding protein 1 (XBP1) splicing in mast cells. (A) RBL-2H3 mast cells were treated with Tg for 15 or 60 min at 37 ° C (n = 4). (B) Densitometry of XBP1 mRNA levels was performed using the ImageJ image analysis software. Results are represented as the mean ± standard error (SE). ***$p < 0.001$ via Dunnett's test.

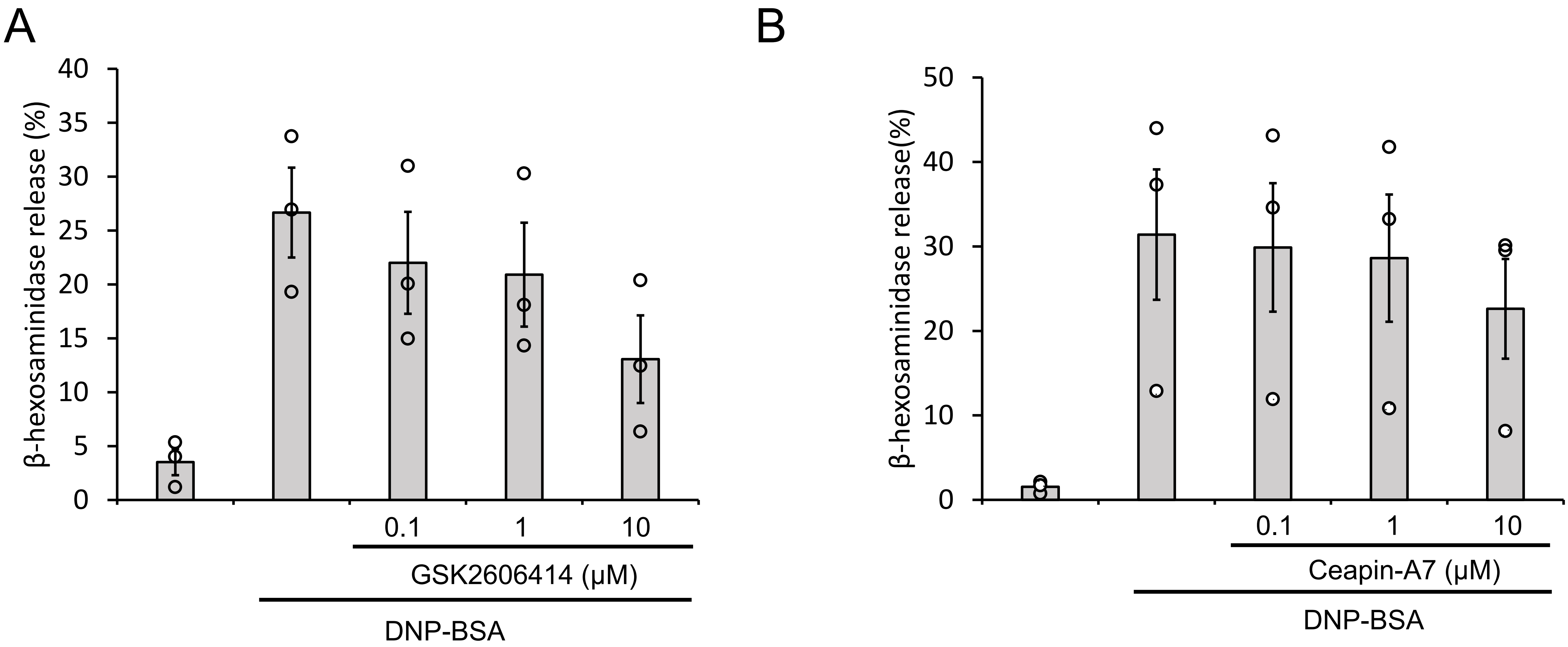

Supplementary figure 2. Protein kinase-like endoplasmic reticulum kinase (PERK) or activating transcription factor-6 (ATF6) inhibitors do not significantly affect antigen-induced mast cell degranulation. (A and B) Bone marrow-derived mast cells (BMMCs) sensitized with anti-DNP IgE antibodies (100 ng/mL) overnight were treated with PERK inhibitor GSK2606414 or ATF6 inhibitor Ceapin-A7 for 30 min at the indicated concentrations and stimulated with antigens (50 ng/mL DNP-bovine serum albumin [BSA]) for 15 min at 37 ° C (n = 3). Results are represented as the mean ± SE.

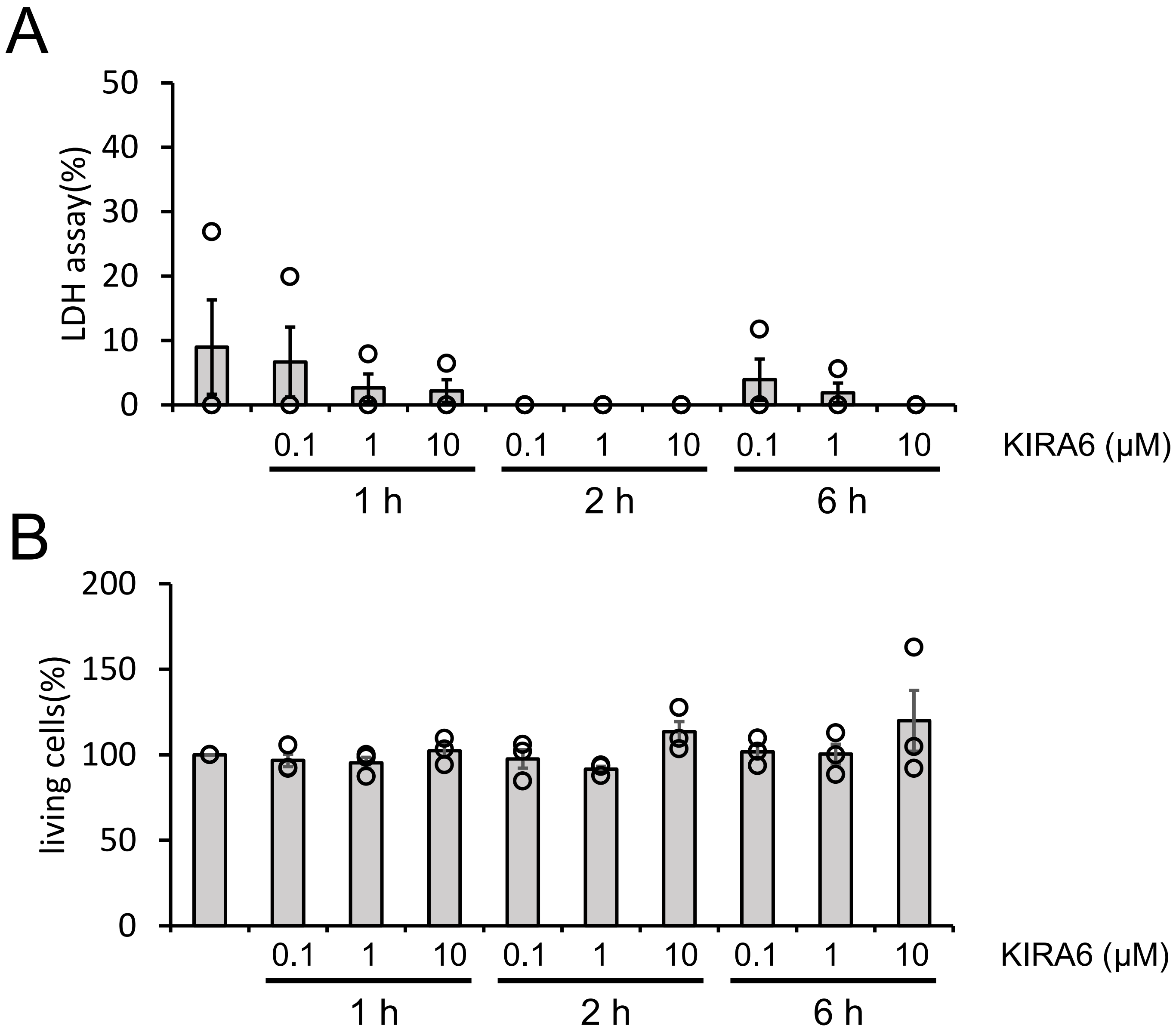

Supplementary figure 3. KIRA6 does not affect the cell death and survival rates of mast cells. (A and B) BMMCs were treated with IRE1α inhibitor KIRA6 at the indicated concentrations for 1, 2, and 6 h at 37 ° C. Lactate dehydrogenase (LDH) release was measured using the Cytotoxicity LDH Assay Kit-WST (n = 3). Live cell count was measured using the cell counting kit-8 (n = 3). Results are represented as the mean ± SE.

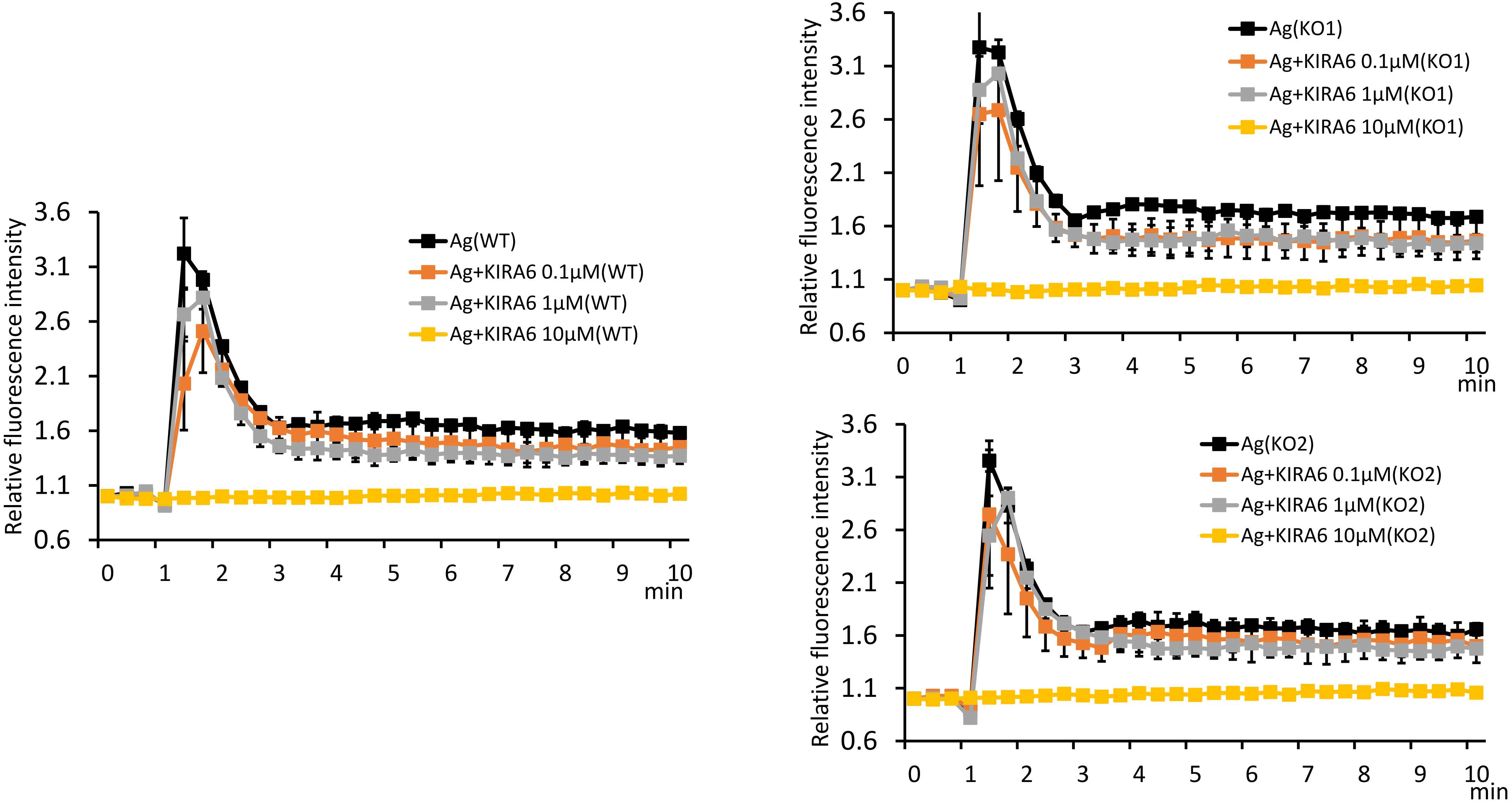

Supplementary figure 4. KIRA6 suppresses antigen-induced intracellular Ca^{2+} mobilization in wild-type (WT), knockout (KO)-1, and KO2 cells. WT, KO1, and KO2 cells sensitized with anti-DNP IgE antibodies (100 ng/mL) overnight were treated with IRE1α inhibitor KIRA6 at the indicated concentrations for 30 min and stimulated with antigens (50 ng/mL DNP-BSA) at 37 ° C (n = 3). Intracellular Ca^{2+} concentrations were measured using Fura 2-AM. Results are represented as the mean ± SE.

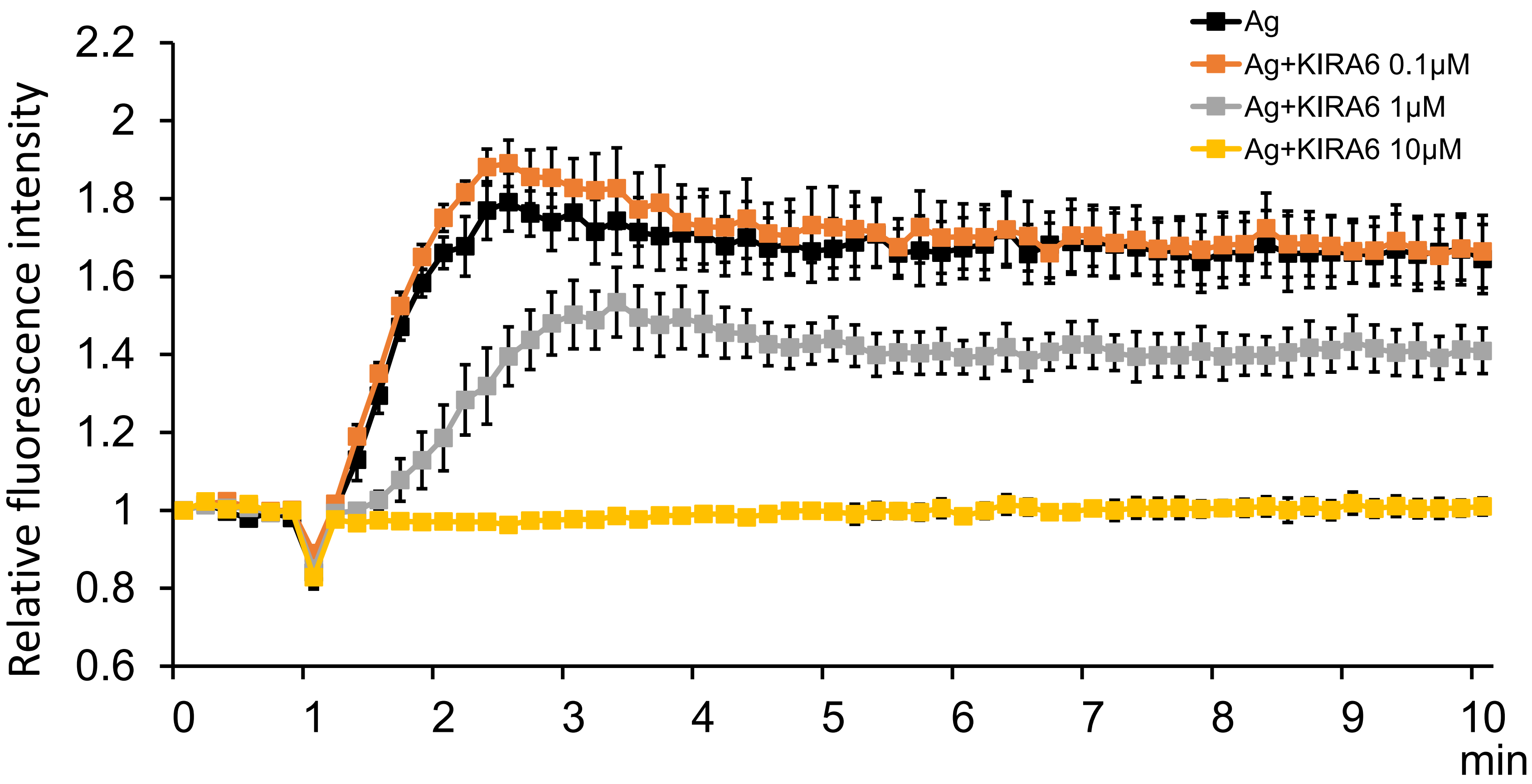

Supplementary figure 5. KIRA6 suppresses antigen-induced intracellular Ca^{2+} mobilization in BMMCs. BMMCs sensitized with anti-DNP IgE antibodies (100 ng/mL) overnight were treated with IRE1α inhibitor KIRA6 at the indicated concentrations for 30 min and stimulated with antigens (50 ng/mL DNP-BSA) at 37 ° C (n = 3). Intracellular Ca^{2+} concentrations were measured using Fura 2-AM. Results are represented as the mean ± SE.
